# Disparities in the Prevalence of Psychiatric Illness in Hawaii's Houseless Population: A Retrospective Chart Review

**DOI:** 10.7759/cureus.56118

**Published:** 2024-03-13

**Authors:** Nicholas E Fancher, Bibek Saha, Shirley Cheng, Angelique Fontaine, Austin Corpuz, Jill Omori

**Affiliations:** 1 Internal Medicine, Los Angeles County University of Southern California Medical Center, Los Angeles, USA; 2 Medicine, University of Hawaii John A. Burns School of Medicine, Honolulu, USA; 3 Family Medicine, University of Hawaii John A. Burns School of Medicine, Honolulu, USA

**Keywords:** ethnicity, mental health disorders, health disparities, homelessness, houselessness

## Abstract

In the State of Hawaii, previous research has suggested that minority groups such as Native Hawaiians and Other Pacific Islanders are disproportionately affected by mental health disorders and have less access to mental health services. The purpose of this study was to determine if similar disparities in the prevalence of psychiatric disorders among different ethnic groups are also present among Hawaii's houseless population. A retrospective chart review of records from one of Oahu's major houseless outreach clinics was performed to gather patient demographics and reported histories of psychiatric diagnoses. Reported disease prevalence rates were compared between larger ethnic categories as well as ethnic sub-groups. Results demonstrated higher rates of certain serious mental illnesses among the houseless; however, several other psychiatric diagnoses were unexpectedly found to be less prevalent than in the general population. Additionally, houseless Pacific Islander groups were unexpectedly found to often have disproportionally lower rates of psychiatric diagnoses despite being identified as high risk by other studies. Overall, our findings may indicate unique ethnic trends in the prevalence of mental health disorders among the houseless in Hawaii or may suggest increased social and/or cultural barriers to diagnosis among certain groups that will require more diligent screening and culturally competent care from providers.

## Introduction

Mental health disorders are highly prevalent conditions responsible for causing the highest proportion of disability-adjusted life years worldwide [1,2]. In the United States, data from the past two decades have shown that ethnic minorities more often receive inadequate mental health care and experience higher rates of chronic mental illness than other ethnic groups [3-5]. Specifically in the State of Hawaii, Native Hawaiians and Other Pacific Islander groups have higher rates of suicide, poor self-reported mental health, and lower engagement with mental health services [6-9]. The houseless are another marginalized group seen to experience a high burden of mental health disorders, especially substance use disorders, schizophrenia spectrum and psychotic disorders, and depressive and anxiety disorders [10,11]. Mental health and houselessness are thought to share a complex, bidirectional relationship. Poor mental health can impair individuals' ability to maintain housing due to difficulties securing a stable income or fulfilling the required responsibilities that accompany housing [12,13]. Once without housing, individuals are more likely to be exposed to stressful and traumatic situations, suffer worse physical health, and struggle to receive consistent healthcare, all of which may exacerbate and perpetuate mental health disorders [14-16]. While both minority status and houselessness individually are known risk factors for poor mental health among Hawaii residents, it remains less clear whether health disparities in psychiatric illness based on ethnicity exist among Hawaii's houseless population.

The Hawaii Houseless Outreach and Medical Education (HOME) Project is one of Hawaii's largest organizations dedicated to providing free healthcare to an almost exclusively houseless population on the island of Oahu. Operating at nine clinic sites with medical students from the John A. Burns School of Medicine (JABSOM) and volunteer physicians, the HOME project serves several hundred houseless individuals each year [17]. In 2010, a review of HOME project chart records by JABSOM researchers found no differences in prevalence rates of multiple mental health disorders between houseless Native Hawaiians and their non-Native Hawaiian houseless counterparts [18]. Since then, the HOME project has continued to serve hundreds of patients each year, providing the opportunity to expand our analysis of mental health disorders among Hawaii's houseless to include additional ethnic groups. Here, we present an updated retrospective review of HOME project chart records focusing on health disparities in psychiatric diagnoses between several houseless ethnic groups in Hawaii.

## Materials and methods

Pilot studies and data abstraction

The University of Hawaii's Institutional Review Board authorized the use of human subjects data for this retrospective chart review. Patient charts were accessed by the four authorized reviewers via the HOME project's secure online electronic medical record system, Practice Fusion. Two pilot studies were completed prior to starting data collection, which involved the creation of a standardized abstraction form in which all demographic (age, sex, ethnicity) and psychiatric (diagnosis of depression, anxiety, etc.) data could be recorded. Each pilot study included a sample of 20 patient charts and confirmed inter-rater reliability between the four independent reviewers. Data was then collected and de-identified using the standardized abstraction form.

Patient charts were excluded if they lacked at least one fully completed visit note or if they only contained visits for tuberculosis skin testing, which is mandatory at certain shelters and thus not a reliable voluntary health behavior. All remaining patients who had been seen at any Oahu HOME clinic site between 05/31/2016 and 01/31/2020 were then included in the study. All patients who met the inclusion criteria were included from all age groups and ethnicities in the initial study population. All demographic data, patient-reported past, and present medical diagnoses, as well as new medical diagnoses established at HOME clinic visits, were collected from patient charts. Psychiatric diagnoses established at HOME clinic sites were based on the DSM-5 diagnostic criteria for anxiety disorders, major depressive disorder (MDD), schizophrenia spectrum and other psychotic disorders (excluding mood disorders with psychotic features), post-traumatic stress disorder (PTSD), bipolar disorder, and attention-deficit/hyperactivity disorder (ADHD). Interviews at HOME clinic sites discussing patient symptoms were initially conducted by medical students before all diagnoses were confirmed with the clinic's licensed supervising physicians.

Ethnic categories

For the purposes of examining health disparities between ethnic groups, patients were placed into one of eight categories based on their self-reported ethnic background: 1) Caucasian - solely "White," "Caucasian," or a descendent of the original peoples of Europe or North Africa. 2) Asian - solely "Asian" or a descendant of the original peoples of Korea, China, Japan, Indonesia, or other Southeast Asian countries. Patients reporting two or more ethnicities that all fell within this category were included in this group. 3) Filipino - solely "Filipino" or a descendant of the original people of the Philippines. 4) Native Hawaiian - solely "Native Hawaiian" or a descendant of the original people of Hawaii. 5) Mixed Native Hawaiian (MNH) - comprised of the three largest mixed ethnicity groups, which included Native Hawaiian ancestry: Native Hawaiian/Caucasian, Native Hawaiian/Chinese, and Native Hawaiian/Filipino. Other mixed ethnicity patients with Native Hawaiian ancestry were not included as sample sizes were too small for adequate sub-group analysis. 6) Other Pacific Islander (OPI) - solely a descendent of the original peoples of Micronesia, Tonga, Samoa, or other Pacific Islands other than Hawaii. Patients reporting two or more ethnicities that all fell within this category were included in this group. Additionally, patients reporting Micronesian ancestry without further specifying a Micronesian sub-group were placed in a "Micronesian, Unspecified" group. 7) African American - solely "Black," "African American," or a descendant of the black racial groups of Africa. 8) Hispanic - solely "Hispanic American," "Latino," or a descendent of Central or South American countries.

All other patients with two or more ethnicities that did not meet the criteria for inclusion in one of the eight primary categories were excluded from our analysis of ethnicity-based health disparities to ensure good generalizability of our study. Sub-group analyses between individual ethnic groups within the eight primary categories were carried out as deemed appropriate. Ethnic sub-groups were included in this portion of our analysis only if they contained at least 12 patients, as groups with smaller sample sizes were deemed too under-powered for meaningful statistical analysis.

Statistical analysis

Descriptive statistics were calculated to summarize patient characteristics. Specifically, means and standard deviations were calculated for continuous variables. For our analyses of disease prevalence by ethnic group, two-tailed Chi-squared tests were performed to compare groups when each contained a sample size of at least ten patients, while two-tailed Fisher's exact tests were used for comparisons involving smaller groups. Simple regression was used to examine associations between age and psychiatric diagnoses. For the purposes of this study, a p-value less than 0.05 was considered statistically significant. All statistical calculations were performed using Microsoft Excel version 16.66.1 (Microsoft, Redmond, Washington) and GraphPad Prism version 9.2.0 (GraphPad Software, La Jolla, California).

## Results

Baseline characteristics

Of a total of 5464 patient charts reviewed, 3407 were either excluded for lacking sufficient data or found to be duplicates and combined. Therefore, 2057 unique patients were ultimately included in our study. Of the included patients, 65.9% were male, 33.9% were female, and 0.2% declined to report a sex. The average age of the entire study population was 50.5 ± 17.4 years, with 91.4% of patients being age 18 or older. The average age for males was 52.1 ± 16.7 years, while the average age for females was 43.9 ± 18.2 years (Table 1). Of the included patients, 23.8% reported a current or past history of psychiatric conditions. Of the patients who reported a history of psychiatric illness, 50.5% had a history of multiple different psychiatric diagnoses. Overall prevalence rates of specific psychiatric conditions are shown in Table 1.

**Table 1:** Patient demographics and reported whole population prevalence of mental health conditions SD, standard deviation ᵃDefined by the DSM-V diagnostic category of the same name which includes schizophrenia, schizophreniform disorder, schizoaffective disorder, delusional disorder, and psychotic disorder not otherwise specified. Does not include mood disorder with psychotic features.

Demographic	n	Percent of population (%)
Male	1356	65.9
Female	697	33.9
No reported sex	4	0.2
	n	Mean ± SD
Age, all patients (years)	2057	50.5 ± 17.4
Age, males (years)	1356	52.1 ± 16.7
Age, females (years)	697	43.9 ± 18.2
Psychiatric diagnosis	n	Prevalence (%)
Attention deficit hyperactivity disorder	32	1.6
Anxiety disorder	109	5.3
Bipolar disorder	122	5.9
Major depressive disorder	156	7.6
Schizophrenia spectrum and other psychotic disordersᵃ	121	5.9
Post-traumatic stress disorder	75	3.6
Multiple psychiatric diagnoses	202	50.5

Sex differences

Males and females did not significantly differ in the reported prevalence rates of ADHD (p=0.745), anxiety disorders (p=0.213), bipolar disorder (p=0.500), MDD (p=0.261), psychotic disorders (p=0.110), or PTSD (p=0.169; Table 2). Males and females also did not differ in the frequency of being diagnosed with multiple psychiatric conditions (p=0.942).

**Table 2:** Sex differences in reported prevalence rates of psychiatric disease * indicates a statistically significant p-value (<.05). All p-values were calculated by a chi-squared test of independence. ᵃ Defined by the DSM-V diagnostic category of the same name, which includes schizophrenia, schizophreniform disorder, schizoaffective disorder, delusional disorder, and psychotic disorder not otherwise specified. Does not include mood disorders with psychotic features.

Psychiatric diagnosis	Men (n)	Women (n)	X^2^ value	p-value
Attention deficit hyperactivity disorder	22	10	0.106	0.745
Anxiety disorder	66	43	1.552	0.213
Bipolar disorder	84	38	0.454	0.5
Major depressive disorder	96	59	1.266	0.261
Schizophrenia spectrum and other psychotic disordersᵃ	88	33	2.557	0.11
Post-traumatic stress disorder	44	31	1.89	0.169
Multiple psychiatric diagnoses	134	68	0.005	0.942

Associations with age

Younger age was found to be significantly associated with a diagnosis of ADHD (p<0.0001). No significant associations were found between patient age and diagnosis of an anxiety disorder, MDD, schizophrenia spectrum, and other psychotic disorders, PTSD, or bipolar disorder.

Ethnic categories

Of the 2057 patients originally included in the study, 936 were unable to be placed in one of the eight major ethnic categories and excluded from our analysis of health disparities. The majority of the excluded patients either declined to report an ethnicity (n=471) or were mixed ethnicity subjects that failed to meet the definition of any of the eight ethnic categories (n=454). Native Americans (n=11) were also excluded as the group's small size was not conducive to meaningful statistical analysis. Of the remaining 1121 patients included in our analysis of health disparities, Caucasians were the largest ethnic group comprising 36.5% of the study population, followed by OPI (24.1%), Asians (9.5%), Native Hawaiians (8.3%), MNH (7.4%), African Americans (5.9%), Filipinos (5.2%), and Hispanics (3.2%) (Table 3). Caucasians were found to have a significantly higher number of individuals with a history of multiple psychiatric diagnoses than Native Hawaiians (p=0.0114). No other differences were found between ethnic groups in rates of individuals with a history of multiple psychiatric diagnoses.

**Table 3:** Demographic data and prevalence rates of psychiatric conditions by major ethnic category NH - Native Hawaiian; MNH - Mixed Native Hawaiian; OPI - Other Pacific Islander; SD - standard deviation; ADHD - attention-deficit hyperactivity disorder ᵃ One patient of the Caucasian ethnic group did not report a sex, representing 0.2% of the group. ᵇ Defined by the DSM-V diagnostic category of the same name, which includes schizophrenia, schizophreniform disorder, schizoaffective disorder, delusional disorder, and psychotic disorder not otherwise specified. Does not include mood disorders with psychotic features.

Demographic variable	Caucasian	OPI	Asian	NH	MNH	African American	Filipino	Hispanic
Population size (n)	409	270	106	93	83	66	58	36
Percent of total population (%)	36.5	24.1	9.5	8.3	7.4	5.9	5.2	3.2
Sex (% Male/Female)	74.6/25.2ᵃ	57.4/42.6	68.9/31.1	43.0/57.0	68.7/31.3	69.7/30.3	69.0/27.3	77.8/22.2
Age (Mean ± SD)	52.0 ± 13.9	36.3 ± 20.4	56.0 ± 13.3	44.0 ± 17.0	50.1 ± 17.1	47.4 ± 17.2	51.9 ± 15.2	48.8 ± 12.5
Psychiatric diagnosis								
Attention deficit hyperactivity disorder (%)	1.7	0.7	0.0	1.1	1.2	1.5	0.0	0.0
Anxiety disorder (%)	11.2	0.7	1.9	3.2	1.2	3.0	6.9	0.0
Bipolar disorder (%)	8.3	1.5	3.8	4.3	3.6	10.6	0.0	11.1
Major depressive disorder (%)	12.5	1.5	6.6	9.7	12.0	7.6	5.2	11.1
Schizophrenia spectrum/psychotic disordersᵇ (%)	5.1	0.7	7.5	4.3	4.8	7.6	6.9	11.1
Post-traumatic stress disorder (%)	6.6	1.1	1.9	0.0	1.2	1.5	0.0	11.1
Multiple psychiatric diagnoses (%)	42.9	28.6	42.1	22.2	37.5	42.9	28.6	41.7

Anxiety disorders

The reported prevalence of anxiety disorders was higher among Caucasians when compared with Asians (p=0.001), Native Hawaiians (p=0.019), MNH (p=0.002), OPI (p=<0.001), African Americans (p=0.045), and Hispanics (p=0.039). Anxiety disorders were also reported at higher rates among Filipinos than OPI (p=0.010; Table 4). On sub-group analysis of the OPI group, Caucasians were found to have a higher reported prevalence of anxiety disorders than Chuukese (p=<0.001), Marshallese (p=0.023), Samoans (p=0.045), and Pacific Islanders reporting two or more ethnicities (p=0.019). Chuukese were additionally found to have a lower reported prevalence of anxiety disorders than Filipinos (p=0.035). On sub-group analysis of the MNH group, Caucasians were found to have a higher reported prevalence of anxiety disorders than the Hawaiian/Caucasian group (p=0.023; Table 5).

**Table 4:** Comparison of reported psychiatric disorder prevalence between major ethnic categories (p-values) NH - Native Hawaiian; MNH - Mixed Native Hawaiian; OPI - Other Pacific Islander All p-values were calculated by a Fisher's exact test unless labeled with a (†), which indicates a Chi-Squared test of Independence was used. * indicates a statistically significant p-value (<.05). ᵃ Defined by the DSM-V diagnostic category which includes schizophrenia, schizophreniform disorder, schizoaffective disorder, etc., as well as psychotic disorder not otherwise specified. Does not include mood disorders with psychotic features.

Attention-deficit hyperactivity disorder	White	Asian	Filipino	NH	MNH	OPI	African American
Asian	0.354						
Filipino	0.604	1.000					
Native Hawaiian	1.000	0.467	1.000				
Mixed Hawaiian	1.000	0.439	1.000	1.000			
Other Pacific Islander	0.323	1.000	1.000	1.000	0.554		
African American	1.000	0.384	1.000	1.000	1.000	0.482	
Hispanic	1.000	1.000	1.000	1.000	1.000	1.000	1.000
Anxiety disorder	White	Asian	Filipino	NH	MNH	OPI	African American
Asian	0.001*						
Filipino	0.494	0.186					
Native Hawaiian	0.019*	0.667	0.429				
Mixed Hawaiian	0.002*	1.000	0.159	0.623			
Other Pacific Islander	<0.001*	0.316	0.010*	0.109	0.554		
African American	0.045*	0.638	0.417	1.000	0.584	0.174	
Hispanic	0.039*	1.000	0.294	0.559	1.000	1.000	0.539
Bipolar Disorder	White	Asian	Filipino	NH	MNH	OPI	African American
Asian	0.144						
Filipino	0.014*	0.298					
Native Hawaiian	0.275	1.000	0.299				
Mixed Hawaiian	0.173	1.000	0.268	1.000			
Other Pacific Islander	<0.001*	0.229	1.000	0.211	0.362		
African American	0.486	0.108	0.014*	0.203	0.109	0.001*	
Hispanic	0.533	0.112	0.019*	0.218	0.197	0.008*	1.000
Major Depressive Disorder	White	Asian	Filipino	NH	MNH	OPI	African American
Asian	0.119						
Filipino	0.125	1.000					
Native Hawaiian	0.595	0.447	0.373				
Mixed Hawaiian	.915†	0.211	0.239	0.635			
Other Pacific Islander	<0.001*	0.014*	0.108	0.133	<0.001*		
African American	0.308	1.000	0.722	0.780	0.422	0.017*	
Hispanic	1.000	0.470	0.422	0.755	1.000	0.008*	0.717
Schizophrenia and Other Psychotic Disordersᵃ	White	Asian	Filipino	NH	MNH	OPI	African American
Asian	0.346						
Filipino	0.535	1.000					
Native Hawaiian	1.000	0.386	0.484				
Mixed Hawaiian	1.000	0.555	0.717	1.000			
Other Pacific Islander	0.002*	0.001*	0.010*	0.040*	0.029*		
African American	0.386	1.000	1.000	0.491	0.510	0.004*	
Hispanic	0.134	0.500	0.477	0.218	0.242	0.002*	0.717
Post-Traumatic Stress Disorder	White	Asian	Filipino	NH	MNH	OPI	African American
Asian	0.061						
Filipino	0.037*	0.540					
Native Hawaiian	0.005*	0.500	1.000				
Mixed Hawaiian	0.066	1.000	1.000	0.472			
Other Pacific Islander	<0.001*	0.624	1.000	0.573	1.000		
African American	0.791	1.000	1.000	0.415	1.000	0.585	
Hispanic	0.301	0.036*	0.019*	0.005*	0.029*	0.004*	0.051

**Table 5:** Comparison of reported anxiety disorder prevalence between ethnic sub-groups (p-values) All p-values were calculated by Fisher's exact test. * Indicates a statistically significant p-value (<.05). ᵃ Sub-group consisting of mixed ethnicity subjects whose ethnicities all fall within the "Asian" category. ᵇ Sub-group consisting of mixed ethnicity subjects whose ethnicities all fall within the "Other Pacific Islander" category.

Other Pacific Islander group	Chuukese	Marshallese	Micronesian, unspecified	Tongan	Samoan	Pacific Islander, otherᵇ
African American	0.221	0.539	1.000	1.000	1.000	1.000
Caucasian	<0.001*	0.023*	0.059	0.093	0.045*	0.019*
Portuguese	1.000	1.000	1.000	1.000	1.000	1.000
Chinese	1.000	1.000	1.000	1.000	1.000	1.000
Japanese	0.339	1.000	1.000	1.000	1.000	1.000
Korean	1.000	1.000	1.000	1.000	1.000	1.000
Vietnamese	1.000	1.000	1.000	1.000	1.000	1.000
Asian, otherᵃ	0.327	1.000	1.000	1.000	1.000	1.000
Filipino	0.035*	0.294	0.297	0.316	0.377	0.203
Native Hawaiian	0.255	0.559	1.000	1.000	1.000	1.000
Hawaiian/Caucasian	1.000	1.000	1.000	1.000	1.000	1.000
Hawaiian/Chinese	1.000	1.000	1.000	1.000	1.000	1.000
Hawaiian/Filipino	0.229	0.379	0.431	0.478	0.539	0.467
Hispanic	1.000	1.000	1.000	1.000	1.000	1.000
Marshallese	1.000					
Micronesian, unspecified	1.000	1.000				
Tongan	1.000	1.000	1.000			
Samoan	0.388	1.000	1.000	1.000		
Pacific Islander, otherᵇ	0.448	1.000	1.000	1.000	1.000	
Asian group	Chinese	Japanese	Korean	Vietnamese	Asian, otherᵃ	
African American	1.000	1.000	1.000	1.000	1.000	
Caucasian	0.377	0.104	0.382	1.000	0.157	
Portuguese	1.000	1.000	1.000	1.000	1.000	
Filipino	1.000	0.645	0.580	1.000	0.646	
Native Hawaiian	1.000	1.000	1.000	1.000	1.000	
Hawaiian/Caucasian	1.000	1.000	1.000	1.000	1.000	
Hawaiian/Chinese	1.000	1.000	1.000	1.000	1.000	
Hawaiian/Filipino	1.000	1.000	1.000	1.000	1.000	
Hispanic	1.000	1.000	1.000	1.000	1.000	
Japanese	1.000					
Korean	1.000	1.000				
Vietnamese	1.000	1.000	1.000			
Asian, otherᵃ	1.000	1.000	1.000	1.000		
Mixed Native Hawaiian group	Hawaiian/ Caucasian	Hawaiian/ Chinese	Hawaiian/ Filipino			
African American	0.539	1.000	1.000			
Caucasian	0.023*	0.094	0.492			
Portuguese	1.000	1.000	1.000			
Filipino	0.294	0.310	1.000			
Hispanic	1.000	1.000	0.379			
Native Hawaiian	0.559	1.000	0.578			
Hawaiian/Chinese	1.000					

Major depressive disorder

MDD was reported at lower rates among OPI than Caucasians (p<0.001), Asians (p=0.014), MNH (p<0.001), African Americans (p=0.017), and Hispanics (p=0.008; Table 4). On sub-group analysis of the OPI category, Chuukese were found to have a lower reported prevalence of MDD than African Americans (p=0.021), Caucasians (p<0.001), Native Hawaiians (p=0.005), Hispanics (p=0.010), and all MNH sub-groups (Hawaiian/Caucasian (p=0.010), Hawaiian/Chinese (p=0.015), Hawaiian Filipino (p=0.011)). Marshallese were also found to have a lower reported prevalence of MDD when compared with Caucasians (p=0.014) and Hawaiian/Filipinos (p=0.0499). Finally, Caucasians were additionally found to have higher reported rates of MDD than Samoans (p=0.029) and Pacific Islanders reporting two or more ethnicities (p=0.030; Table 6).

**Table 6:** Comparison of reported major depressive disorder prevalence between ethnic sub-groups (p-values) All p-values were calculated by a Fisher's exact test. (*) indicates a statistically significant p-value (<.05). (a) Sub-group consisting of mixed ethnicity subjects whose ethnicities all fall within the "Asian" category. (b) Sub-group consisting of mixed ethnicity subjects whose ethnicities all fall within the "Other Pacific Islander" Category.

Other Pacific Islander Group	Chuukese	Marshallese	Micronesian, unspecified	Tongan	Samoan	Pacific Islander, otherᵇ
African American	0.021*	0.159	0.663	0.319	0.398	0.444
Caucasian	<0.001*	0.014*	0.233	0.096	0.029*	0.030*
Portuguese	1.000	1.000	1.000	1.000	1.000	1.000
Chinese	0.140	0.250	0.505	0.333	0.368	0.426
Japanese	0.339	1.000	1.000	1.000	1.000	1.000
Korean	1.000	1.000	1.000	1.000	1.000	1.000
Vietnamese	1.000	1.000	1.000	1.000	1.000	1.000
Asian, otherᵃ	0.003*	0.054	0.214	0.077	0.081	0.099
Filipino	0.082	0.284	1.000	0.552	0.626	0.677
Native Hawaiian	0.005*	0.061	0.449	0.201	0.164	0.203
Hawaiian/Caucasian	0.010*	0.115	0.370	0.143	0.161	0.193
Hawaiian/Chinese	0.015*	0.064	0.326	0.235	0.117	0.149
Hawaiian/Filipino	0.011*	0.0499*	0.303	0.101	0.092	0.117
Hispanic	0.010*	0.115	0.370	0.143	0.161	0.193
Marshallese	1.000					
Micronesian, unspecified	0.282	0.446				
Tongan	1.000	1.000	1.000			
Samoan	0.388	1.000	1.000	1.000		
Pacific Islander, otherᵇ	0.199	0.526	1.000	1.000	1.000	
Asian Group	Chinese	Japanese	Korean	Vietnamese	Asian, otherᵃ	
African American	1.000	0.412	0.580	1.000	0.318	
Caucasian	1.000	0.069	0.235	1.000	0.795	
Portuguese	1.000	1.000	1.000	1.000	1.000	
Filipino	0.537	1.000	1.000	1.000	0.253	
Native Hawaiian	1.000	0.280	0.602	1.000	0.533	
Hawaiian/Caucasian	1.000	0.194	0.566	1.000	1.000	
Hawaiian/Chinese	1.000	0.292	0.540	1.000	1.000	
Hawaiian/Filipino	1.000	0.135	0.267	1.000	1.000	
Hispanic	1.000	0.194	0.566	1.000	1.000	
Japanese	0.426					
Korean	0.462	1.000				
Vietnamese	1.000	1.000	1.000			
Asian, otherᵃ	1.000	0.103	0.304	1.000		
Mixed Native Hawaiian Group	Hawaiian/ Caucasian	Hawaiian/ Chinese	Hawaiian/ Filipino			
African American	0.717	0.679	0.407			
Caucasian	1.000	1.000	0.751			
Portuguese	1.000	1.000	1.000			
Filipino	0.422	0.259	0.338			
Hispanic	1.000	1.000	1.000			
Native Hawaiian	0.755	0.716	0.697			
Hawaiian/Chinese	1.000					
Hawaiian/Filipino	1.000	1.000				

Schizophrenia spectrum and other psychotic disorders

The reported prevalence of schizophrenia spectrum and other psychotic disorders was lower among OPI when compared with Caucasians (p=0.002), Asians (p=0.001), Filipinos (p=0.010), Native Hawaiians (p=0.040), MNH (p=0.029), African Americans (p=0.004), and Hispanics (p=0.002; Table 4). On sub-group analysis of the OPI category, Chuukese were found to have lower reported rates of psychotic disorders than the Chinese (p=0.0499) and Hispanic (p=0.049) groups. The reported prevalence of psychotic disorders was also lower among Pacific Islanders reporting two or more ethnicities when compared with Chinese (p=0.026), Koreans (p=0.034), and Hispanics (p=0.018; Table 7).

**Table 7:** Comparison of reported schizophrenia and other psychotic disorders prevalence between ethnic sub-groups (p-values) Defined by the DSM-V diagnostic category of the same name, which includes schizophrenia, schizophreniform disorder, schizoaffective disorder, delusional disorder, and psychotic disorder not otherwise specified. Does not include mood disorders with psychotic features. All p-values were calculated by Fisher's exact test. * Indicates a statistically significant p-value (<.05). ᵃ Sub-group consisting of mixed ethnicity subjects whose ethnicities all fall within the "Asian" category. ᵇ Sub-group consisting of mixed ethnicity subjects whose ethnicities all fall within the "Other Pacific Islander" Category.

Other Pacific Islander group	Chuukese	Marshallese	Micronesian, unspecified	Tongan	Samoan	Pacific Islander, otherᵇ
African American	0.100	0.420	0.319	0.319	0.398	0.059
Caucasian	0.226	1.000	0.384	0.621	0.717	0.092
Portuguese	1.000	1.000	1.000	1.000	1.000	1.000
Chinese	0.0499*	0.150	0.081	0.105	0.102	0.026*
Japanese	1.000	1.000	1.000	1.000	1.000	0.388
Korean	0.065	0.186	0.101	0.129	0.129	0.034*
Vietnamese	0.101	0.192	0.121	0.143	0.152	0.063
Asian, otherᵃ	0.102	0.614	0.247	0.268	0.312	0.050
Filipino	0.168	0.646	0.297	0.316	0.377	0.055
Native Hawaiian	0.384	1.000	0.572	0.580	0.664	0.155
Hawaiian/Caucasian	0.550	1.000	1.000	1.000	1.000	0.375
Hawaiian/Chinese	0.156	0.562	0.210	0.490	0.275	0.084
Hawaiian/Filipino	0.408	1.0000	0.431	0.478	0.529	0.268
Hispanic	0.039*	0.357	0.122	0.143	0.161	0.018*
Marshallese	0.550					
Micronesian, unspecified	1.000	1.000				
Tongan	1.000	1.000	1.000			
Samoan	1.000	1.000	1.000	1.000		
Pacific Islander, otherᵇ	1.000	0.375	1.000	1.000	0.439	
Asian group	Chinese	Japanese	Korean	Vietnamese	Asian, otherᵃ	
African American	0.293	0.412	0.600	0.307	1.000	
Caucasian	0.138	0.710	0.177	0.200	0.434	
Portuguese	1.000	1.000	1.000	1.000	1.000	
Filipino	0.272	0.645	0.330	0.292	1.000	
Native Hawaiian	0.139	1.000	0.176	0.194	0.398	
Hawaiian/Caucasian	0.150	1.000	0.186	0.192	0.614	
Hawaiian/Chinese	0.582	0.557	0.609	0.371	1.000	
Hawaiian/Filipino	0.279	1.000	0.547	0.289	1.000	
Hispanic	0.631	0.194	1.000	0.427	1.000	
Japanese	0.139					
Korean	1.000	0.173				
Vietnamese	1.000	0.184	1.000			
Asian, otherᵃ	0.587	0.351	0.611	0.356		
Mixed Native Hawaiian group	Hawaiian/ Caucasian	Hawaiian/ Chinese	Hawaiian/ Filipino			
African American	0.420	1.000	1.000			
Caucasian	1.000	0.636	1.000			
Portuguese	1.000	1.000	1.000			
Filipino	0.646	1.000	1.000			
Hispanic	0.357	1.000	0.640			
Native Hawaiian	1.000	0.606	1.000			
Hawaiian/Chinese	0.562					
Hawaiian/Filipino	1.000	1.000				

Post-traumatic stress disorder

The reported prevalence of PTSD was higher among Caucasians when compared with Filipinos (p=0.037), Native Hawaiians (p=0.005), and OPI (p<0.001). Hispanics were also found to have a higher reported prevalence of PTSD than Asians (p=0.036), Filipinos (p=0.019), Native Hawaiians (p=0.005), MNH (p=0.029), and OPI (p=0.004; Table 4). On sub-group analysis of the OPI category, Chuukese were found to have lower reported rates of PTSD than Caucasians (p=0.013) and Hispanics (p=0.010). The reported prevalence of PTSD was also lower among Samoans than Hispanics (p=0.032). The full data set for PTSD ethnic sub-group analyses is available on request from the authors.

Bipolar disorder

The reported prevalence of bipolar disorder was lower among both OPI and Filipinos when compared with Caucasians (p<0.001 and p=0.014), African Americans (p=0.001 and p=0.014), and Hispanics (p=0.008 and p=0.019), respectively (Table 4). On sub-group analysis of the OPI category, Chuukese were found to have lower reported rates of bipolar disorder than African Americans (p=0.004), Caucasians (p=0.005), and Hispanics (p=0.010). The full data set for bipolar disorder ethnic sub-group analyses is available on request from the authors.

Additional data on differences in the prevalence of ADHD among the major ethnic categories is summarized in Table 4.

## Discussion

The burden of mental health disorders has been growing on a national level, and the state of Hawaii has been found to have some of the highest rates of untreated mental illness in the country [19]. This crisis may be even more impactful for some of Hawaii's most vulnerable populations, such as minority groups and the houseless. For instance, suicide rates among Native Hawaiians aged 25-44 have been cited at 92 per 100,000, compared with the overall rate of 76 per 100,000 in Hawaii [6]. Hawaii's houseless were found to have an age- and sex-adjusted rate of admission to the state psychiatric hospital of 105/1000 person-years compared with the overall state rate of 0.8/1000 person-years [20]. This retrospective chart review, to our knowledge, is the most comprehensive study to date focused on whether ethnic differences in psychiatric illness observed in Hawaii's general population are also seen among Hawaii's houseless.

About two-thirds of our study population identified as male, largely consistent with recent surveys of Oahu's houseless, which found 56% to identify as male [21]. This gender imbalance among the houseless is thought to be due to higher houselessness rates among veterans and lower utilization of mental health services by men, among other factors [22,23]. Female patients were, on average, younger than males by about eight years, which may be related to women more often being seen for obstetric reasons or being seen alongside their children.

Multiple psychiatric conditions were reported at lower rates in our houseless population when compared to the general Hawaii or national population. These included lower rates of MDD (7.6% vs. 8.8%) and anxiety (5.3% vs. 8.0%) in our houseless cohort compared to the general Hawaii population [24]. Although Hawaii-specific prevalence data was unavailable for ADHD and PTSD, reported rates in our houseless cohort for each were lower than the national average (1.6% vs. 4.4%, 3.6% vs 6.8%, respectively) [25,26]. However, reported rates were instead higher among our houseless population for bipolar disorder (5.9% vs. 2.3%) when compared to the general Hawaii population, and for schizophrenia spectrum and other psychotic disorders (5.9% vs. 3.1-3.5%) when compared to the general US population [27,28].

Overall, our findings are unexpected, as previous work has shown nearly all psychiatric disorders to be more prevalent among the houseless [29,30], yet the pattern in our results may be explained by medical underdiagnosis. Houseless individuals have much lower access to routine healthcare services, especially mental health services known to be limited in Hawaii [19]. Furthermore, these individuals may be subject to suboptimal psychiatric care due to provider stigma surrounding their houseless status [31]. As a result, less clinically evident psychiatric conditions such as ADHD or anxiety may remain undiagnosed. However, disorders such as bipolar I or schizophrenia that involve psychosis and/or hallucinations are more likely to lead to hospitalization and be clinically evident to providers. These disorders may also lead to a higher degree of social or occupational dysfunction in the setting of inadequate treatment, predisposing individuals to becoming houseless.

A number of ethnic differences were found in our analysis of psychiatric conditions in this houseless cohort. The prevalence of bipolar disorder in our population was found to be lower among both OPI and Filipinos than Caucasians, African Americans, and Hispanics, which is consistent with national studies that have identified Asians and Other Pacific Islanders to have lower rates of bipolar disorder than Whites [32]. African Americans are typically identified as experiencing the highest rates of bipolar disorder in the US in contrast with our findings; however, this may be related to the relatively small size of the African American population in the State of Hawaii overall [33,34]. We identified lower rates of psychotic disorders among OPI in our population when compared with all other ethnic categories. Previous literature on schizophrenia spectrum and other psychotic disorders in the general Hawaii population instead identified higher rates among Asians but did not find similar differences for Native Hawaiians and OPI [35,36].

Caucasians were identified as reporting higher rates of anxiety than every other ethnic category except for Filipinos in our population. This finding has been supported in the literature for both the general Hawaii population as well as the national population [37,38]. PTSD was found to be reported at higher rates among Caucasians and Hispanics than multiple other ethnic groups in our population, including Native Hawaiians, OPI, and Filipinos. These findings may be seen to conflict with prior work that has identified higher rates of trauma exposure among Native Hawaiians than other ethnic groups in Hawaii [39]. National surveys have partially supported our findings on PTSD, where both Caucasians and African Americans, but not Hispanics, report the highest rates of PTSD [40]. The OPI category was identified as having a lower reported prevalence of MDD than several other ethnic groups, with similar findings for multiple OPI sub-groups. These results conflict with previous findings that Pacific Islanders experience depression at significantly higher rates than Asians in Hawaii [41].

Although some ethnic differences observed in the general Hawaii or US population were also seen among our houseless cohort, several other ethnic trends were divergent in our population. OPI and multiple of the categories' sub-groups had notably low prevalence rates of psychiatric diagnoses despite being identified as high risk by other studies. This may be partially explained by Pacific Islander's low rates of engagement with mental health services; multiple studies have observed that in addition to having lower access to care, many Pacific Islanders choose to delay or avoid mental health disorder treatment, possibly due to cultural stigma against mental illness among their communities [8,42,43]. OPI patients at HOME clinic sites, thus, may be less willing to report psychiatric symptoms than their peers.

The burden of multiple psychiatric diseases, including anxiety and PTSD, was notably higher for Caucasians than many other ethnicities in our houseless population. Similarly, we found Caucasians to be more likely to have multiple psychiatric diagnoses than Native Hawaiians. The literature supports higher lifetime prevalence rates of many mental disorders among Caucasians compared to other ethnicities in the US but identifies higher chronicity of mental illness among minority groups, a characteristic we did not elucidate [4,38,44]. Some have theorized that lower rates of certain psychiatric diagnoses among US minority groups, including anxiety and depressive disorders, may be due to a protective effect of a strong ethnic identity and, in some cases, higher religious participation [4,45]. However, others have proposed that Caucasians instead experience fewer barriers to being diagnosed than other groups (i.e., easier access to care, less provider bias) [46]. While we might expect houseless Caucasians to similarly experience issues with insurance or financial resources, transportation, or medication storage as their houseless minority peers, houseless Caucasians may still encounter fewer issues with provider bias, language barriers, and poor cultural competency while receiving healthcare [47]. Clinicians' conscious and unconscious biases towards minority patients have historically been known to affect multiple aspects of psychiatric care in particular [48], and the impact of these biases among HOME clinic providers is important to consider.

This study had several limitations. Firstly, the collection of past medical diagnoses during clinic visits was reliant on voluntary reporting and accurate recall by HOME clinic patients who may have limited health literacy or English proficiency. Clinic providers as well may be more likely to struggle with psychiatric histories when confronted with conflicting interpretations of such topics between themselves and patients. As mentioned previously, cultural or linguistic barriers can have a major effect on the therapeutic relationship, and this challenge may be magnified in the context of sensitive topics like mental health [49]. The lack of guaranteed follow up visits with patients can also limit providers' abilities to accurately assign psychiatric diagnoses that require quantifying the duration of symptoms. Additionally, our sample of Oahu's houseless population was confined to HOME clinic sites, which often are located in or near shelters, possibly skewing our surveyed population towards sheltered over unsheltered houseless individuals. The retrospective nature of our study was another limitation, as ultimately, a considerable number of patient charts needed to be excluded for failing to contain sufficient data. Although few associations were found between sex, age, and psychiatric illness, it should be noted that our analyses of ethnic differences in disease prevalence were not controlled for sex or age. The influence of physical comorbidities and substance use on the development and persistence of psychiatric disease, as well as factors protective against psychiatric disease (ie. support networks), could have been considered as well.

The largest limitation of our study was the need to exclude patients of mixed ethnicity who failed to meet the criteria for our eight major ethnic categories, as our results will have low generalizability for this substantial subset of the houseless population. Previous studies have also suggested that multiethnic individuals may be especially predisposed to poor mental health, possibly related to struggles with their racial identity or due to discrimination by others [50]. By excluding the majority of multiethnic patients, we potentially excluded a disproportionately high number of patients with mental health issues from our analyses of ethnic differences. It also may have limited our ability to compare our results with other studies of the general Hawaii or national population who defined their ethnic categories differently, such as grouping many mixed ethnicity subjects into categories like "Part-Native Hawaiian" based solely on the presence of any Native Hawaiian ancestry. However, this is also a strength of our study, as by excluding this subset of mixed ethnicity patients, we are able to increase the generalizability of our results for the eight ethnic categories that comprise the majority of Oahu's houseless population. Another notable strength of our study was the use of a standardized abstraction form and execution of multiple pilot studies prior to data collection, which allowed us to maximize inter-rater reliability and minimize measurement bias.

In addition to revealing high prevalence rates of certain serious mental disorders among the houseless in Hawaii, our study also highlights possible shortcomings in identifying psychiatric disorders among certain ethnic groups. Houselessness is known to be highly associated with the development and persistence of mental illness, and as such, our findings of lower prevalence rates of several psychiatric disorders serve as a red flag for widespread underdiagnosis at HOME clinic sites [10,51]. The houseless face a myriad of challenges on a daily basis, and as such, mental health issues like persistent feelings of anxiety or depression may not be one of their chief concerns. Providers working with the houseless should thus consider utilizing efficient mental health screening tools such as the Patient Health Questionnare-2 (PHQ-2) for depression, the Generalized Anxiety Disorder 2-item (GAD-2) for anxiety, and the Primary Care PTSD Screen for DSM-5 (PC-PTSD-5) routinely, when possible. Since the time period surveyed for this study, the HOME clinic has already begun implementing multiple of the aforementioned screening tools as part of routine assessments. Managing psychiatric illness can also be a challenge with houseless individuals who may face difficulties holding on to medications, and as such, providers should consider alternative therapies like long-acting depot injections when feasible.

Our study has also highlighted possible cultural barriers to seeking, accepting, and delivering mental health care treatment among Pacific Islander populations, such as how Micronesian and Polynesian groups are more likely to attribute mental illness to moral failings of the person or their family [43]. Strategies to overcome these barriers have been discussed by many experts in the field [49,52,53], and the HOME project and its affiliates have already made some progress on developing solutions. By better integrating mental health care into a general, primary care setting like a HOME clinic, therapeutic relationships can first be established via less stigmatized medical care before more sensitive topics are addressed. This may also be a more practical model for mental health care in a limited resource setting [52], such as caring for the houseless. Additionally, as suggested by Marsella [53], a connection of mental health care with etho-cultural community resources and services can strengthen the effectiveness of culturally competent care. Providers with HOME clinics are also often involved with JABSOM's other outreach projects on Oahu, including youth engagement at shelters and providing health education in public schools. These deeper connections with the community can allow providers to develop their cultural competence and better integrate the psychiatric care they provide with patients' own social support networks.

It should be noted that substance use disorders are another highly prevalent group of mental health disorders among the houseless [10] that are not specifically addressed in this work. While conducting this current chart review, the authors recognized the importance and unique nature of these disorders and conducted a separate in-depth analysis of several substance use disorders among our houseless population. These findings are reported in a separate study to allow for a more focused and comprehensive discussion of their significance.

## Conclusions

In conclusion, we present an updated retrospective chart review of one of Oahu's largest houseless outreach programs focusing on psychiatric disorders across several ethnic groups. Certain ethnic groups among the general population known to be at higher risk of specific psychiatric diseases were shown to be similarly predisposed among Hawaii's houseless, yet for several conditions, our findings for this houseless population diverged from the literature, possibly due to widespread underdiagnosis and cultural barriers to mental health care. We hope this study can help inform providers about the relationships between ethnicity and mental health and reinforce the need for increased screening and culturally sensitive care. Future work is warranted to study the prevalence of mental health disorders among Hawaii’s houseless prospectively utilizing standardized screening tools. Additionally, future studies should investigate different approaches to engaging with Native Hawaiians and OPI on the topic of mental health in a manner that minimizes stigma and aligns with their cultural beliefs and social support network.
